# Cytotaxonomy and molecular phylogeny of the genus *Cerapanorpa* Gao, Ma & Hua, 2016 (Mecoptera: Panorpidae)

**DOI:** 10.1038/s41598-017-04926-9

**Published:** 2017-07-03

**Authors:** Ying Miao, Na Ma, Bao-Zhen Hua

**Affiliations:** 1 State Key Laboratory of Crop Stress Biology for Arid Areas, Key Laboratory of Plant Protection Resources and Pest Management, Ministry of Education, Northwest A&F University, Yangling, Shaanxi 712100 China; 2 School of Agricultural Engineering, Henan Key Laboratory of Insect Biology in Funiu Mountain, Nanyang Normal University, Nanyang, Henan 473061 China

## Abstract

The species of the genus *Cerapanorpa* Gao, Ma & Hua, 2016 (Mecoptera: Panorpidae) are characterized mainly by the presence of a finger-like anal horn on tergum VI of males and are distributed in the Oriental and eastern Palearctic regions. Herein, we investigated the pachytene banding patterns and reconstructed the Bayesian time-calibrated tree of some species of *Cerapanorpa*. All species examined display achiasmate meiosis and the same meiformula 2*n* = 42 + X0, reconfirming the monophyly of *Cerapanorpa*. The great variations in the size and number of heterochromatic bands suggest that they are reliable traits for species delimitation in *Cerapanorpa*. The existence of natural C-banding polymorphism indicates that chromosomal rearrangements likely have contributed to the diversification of chromosomal bands in *Cerapanorpa*. The closely related species of *Cerapanorpa* are reconfirmed to be evolutionarily independent entities by cytogenetic and molecular data. The divergence time estimated from the BEAST analysis shows that *Cerapanorpa* likely originated in the period from the Rupelian (30.7 Ma) to the Burdigalian (19.9 Ma), and most diversification occurred from the Burdigalian to the Piacenzian (17.4–2.8 Ma) in the Neogene. Our data suggest that chromosome rearrangements likely play a significant role in the speciation of *Cerapanorpa*.

## Introduction

Cytogenetic studies can reveal both structural and functional homologies among taxa due to their evolutionary conservation^1–3^. Major structural chromosomal rearrangements are often associated with cytogenetically detectable heterochromatic regions composed of repetitive DNA, and frequently appear in the heterochromatin-euchromatin borders^4–6^. Therefore, comparisons of chromosome banding patterns are able to provide useful information of evolutionary relationships among species and reveal variations in karyotypes that are involved in speciation^7–9^. Pachytene bivalents with hypotonic shock treatment can be used as an alternative to mitotic chromosomes in insects^10^. The well-spread bivalents may reveal accurate bands using various banding methods. Such studies have been conducted in many insect groups, such as Coleoptera^11^, Diptera^12^, Hemiptera^13^, Hymenoptera^14^, Lepidoptera^15^, Odonata^16^, and Orthoptera^17^, but were still lacking in Mecoptera.

The family Panorpidae is commonly known as scorpionflies with the greatest taxonomic complexity in Mecoptera and currently consists of seven genera^18–23^. The genus *Cerapanorpa* Gao, Ma & Hua, 2016 comprises 22 species, which occur in the Oriental and eastern Palearctic regions. The species of *Cerapanorpa* are closely similar in appearance, and are characterized mainly by the presence of a single finger-like anal horn on the posterior margin of tergum VI in the males^20^. Conspicuous genital diversity was found in the closely related *Cerapanorpa* taxa that are otherwise morphologically very similar^24^. However, the internal anatomy is relatively conserved compared with genital features, and exhibits a low variability at the specific level in *Cerapanorpa* ^25, 26^. Recently, the analysis of mitochondrial and nuclear genes led to a molecular phylogeny of Panorpidae, providing noteworthy information on *Cerapanorpa* at the generic level^27^, but cytogenetic information is still very limited in *Cerapanorpa*.

To date, only two species of *Cerapanorpa* have been cytogenetically investigated^28^. *Cerapanorpa emarginata* (Cheng, 1949) and *Cerapanorpa dubia* (Chou & Wang, 1981) were found to have the lowest chromosome number (2*n* = 39) in Panorpidae and similar cytogenetic features to previous records of panorpids, which have an X0 (♂)/XX (♀) sex determination mechanism and achiasmate meiosis^29–31^.

In this investigation, we successfully obtained the C-bands on pachytene bivalents of seven species in *Cerapanorpa*. Our results may shed light on the potential significance of cytogenetic data in species delimitation of the genus. The speciation process of *Cerapanorpa* is briefly discussed in the context of the molecular phylogeny of Panorpidae.

## Methods

### Biological materials

Male adults were collected from a variety of sampling sites in the Qinling and Bashan Mountains of Shaanxi Province, central China from June to August (2013–2015), including *Cerapanorpa brevicornis* (Hua & Li, 2007), *Cerapanorpa byersi* (Hua & Huang, 2007), *C*. *dubia*, *Cerapanorpa nanwutaina* (Chou, 1981), *Cerapanorpa obtusa* (Cheng, 1949), *Cerapanorpa protrudens* Gao, Ma & Hua, 2016, and *Cerapanorpa sinuata* Gao, Ma & Hua, 2016 (see Supplementary Table S1). Specimens used for DNA extraction were stored in absolute ethanol at −20 °C. Photographs of male genitalia were taken with a QImaging Retiga-2000R Fast 1394 Scientific CCD Camera (QImaging, Surrey, Canada) attached to a Nikon SMZ1500 microscope (Nikon, Tokyo, Japan) and were stacked with Syncroscopy Auto-Montage software (Syncroscopy, Cambridge, UK).

### Chromosome preparation, C-banding and statistical analysis

For cytogenetic analysis, the testes of live adults were dissected rapidly in Ringer’s solution and were then submerged in fresh hypotonic KCl solution (0.045 M) for 30 min at room temperature^10^. After a short fixation of 30–40 s in acetic-ethanol (1:3, v/v), the testes were transferred to a drop of 45% acetic acid on glass microscope slides and torn into small pieces. Then the slides were air-dried for 24 h^32^.

C-banding followed the methods described by King^33^. Air-dried slides were placed in HCl solution (0.2 N) for 30 min at room temperature, rinsed in distilled water and dried. The slides were then placed in saturated Ba(OH)_2_ solution at 60 °C for 3–6 min, dipped briefly in HCl and rinsed again in distilled water. Afterwards, the slides were placed in Sørensen’s phosphate buffer (pH 7.0) at 65 °C for 30 min, rinsed in distilled water and stained in 5% Giemsa for 15 min. The slides were then rinsed in distilled water and dried. Photographs were taken with a Nikon DS-Fil digital camera mounted on a Nikon Eclipse 80i microscope (Nikon, Tokyo, Japan).

Ten C-banded meiotic cells at the condensation stage were used to measure the lengths of bivalents. The captured images were quantified using the Microscope Imagining Software NIS-Element D 3.22 (Nikon, Tokyo, Japan). All bivalents were identified based on heterochromatin pattern and their relative lengths. For each bivalent, the mean and standard deviations of relative length (RL = 100 × absolute length/total length of the haploid complement) were calculated using a Microsoft Excel spreadsheet (Table 1). The relative lengths were calculated as a percentage of the total bivalent length of the diploid set without sex chromosome, because of the morphological variation of the sex chromosome during the condensation stage.

**Table 1:** Measurement of pachytene bivalents of seven species in *Cerapanorpa*. RL = relative length; SD = standard deviation.

Bivalent No.^a^	*C*. *obtusa*		*C*. *nanwutaina*		*C*. *sinuata*		*C*. *protrudens*		*C*. *dubia*		*C*. *byersi*		*C*. *brevicornis*	
	RL	SD	RL	SD	RL	SD	RL	SD	RL	SD	RL	SD	RL	SD
1	3.34	0.08	3.78	0.40	3.42	0.24	3.21	0.35	3.30	0.13	3.27	0.06	3.89	0.07
2	3.53	0.09	3.90	0.32	3.63	0.21	3.34	0.24	3.69	0.14	3.98	0.13	3.96	0.04
3	3.86	0.10	4.03	0.25	3.87	0.14	3.91	0.27	3.91	0.11	3.77	0.02	4.06	0.06
4	4.08	0.13	4.13	0.24	4.03	0.12	4.04	0.23	4.08	0.21	4.07	0.03	4.19	0.10
5	4.15	0.09	4.19	0.03	4.29	0.06	4.15	0.08	4.22	0.12	4.23	0.13	4.32	0.02
6	4.24	0.05	4.23	0.04	4.63	0.10	4.38	0.06	4.30	0.10	4.35	0.06	4.45	0.17
7	4.33	0.08	4.33	0.08	4.37	0.10	4.21	0.10	4.39	0.12	4.44	0.04	4.54	0.18
8	4.49	0.09	4.48	0.09	4.44	0.10	4.61	0.13	4.43	0.13	4.50	0.01	4.67	0.07
9	4.59	0.08	4.59	0.12	4.55	0.10	4.70	0.07	4.54	0.07	4.81	0.06	4.72	0.07
10	4.65	0.09	4.64	0.06	4.71	0.10	4.74	0.07	4.58	0.10	4.58	0.02	4.77	0.05
11	4.73	0.08	4.68	0.07	4.78	0.12	4.77	0.04	4.79	0.10	4.70	0.08	4.79	0.05
12	4.87	0.13	4.72	0.04	4.90	0.13	4.79	0.06	4.71	0.07	4.85	0.07	4.88	0.06
13	5.02	0.12	4.75	0.04	5.14	0.14	4.85	0.04	4.84	0.16	4.93	0.04	4.84	0.08
14	5.23	0.14	4.84	0.17	5.18	0.13	4.93	0.08	4.90	0.21	5.04	0.04	4.92	0.06
15	5.06	0.12	5.05	0.17	5.32	0.14	5.29	0.11	5.10	0.24	5.14	0.02	4.98	0.08
16	5.12	0.14	5.30	0.11	4.13	0.14	5.09	0.10	5.19	0.14	5.24	0.00	5.09	0.08
17	5.27	0.14	5.60	0.08	5.00	0.08	5.40	0.07	5.43	0.10	5.21	0.02	5.16	0.10
18	5.92	0.16	5.92	0.01	5.95	0.18	5.82	0.01	5.49	0.13	5.40	0.03	5.49	0.09
19	5.76	0.15	5.45	0.08	5.53	0.17	5.49	0.05	5.58	0.12	5.44	0.04	5.22	0.11
20	5.58	0.15	5.78	0.05	5.70	0.16	6.12	0.12	5.92	0.17	5.68	0.10	5.67	0.27
21	6.25	0.22	6.21	0.22	6.63	0.07	6.14	0.23	6.61	0.47	6.11	0.33	5.92	0.29

^a^Bivalents were ordered according to their C-banding pattern as in Fig. 2.

### DNA extraction and sequencing

We extracted genomic DNA for 10 individuals of *C*. *brevicornis*, eight individuals of *C*. *obtusa*, six individuals of *C*. *dubia*, *C*. *nanwutaina*, *C*. *protrudens* and *C*. *sinuata*, and five individuals of *C*. *byersi* as described by Hu *et al*.^27^. Fragments of one nuclear gene, 28S rRNA, and two mitochondrial genes, cytochrome c oxidase subunit I and II (*cox1* and *cox2*), were amplified using the primer pairs 28S rD3.2a and 28S rD4.2b^34^, C1-J-1718 and C1-N-2329^35^, and COII-F-leu and COII-R-lys^34^, respectively. Polymerase chain reaction (PCR) was performed using 12.5 µL CWBIO 2 × Taq MasterMix, 8.5 µL sterile distilled H_2_O, a pair of 10 µM primers (1 µL each), and 2 µL DNA template in a final volume of 25 µL. The amplification reaction included 5 min initial denaturation at 95 °C, followed by 35 cycles of denaturation at 94 °C for 30 s, annealing at 50.5 °C (for *cox1*) for 30 s, and extension at 72 °C for 1 min, with a final extension at 72 °C for 7 min. The reaction conditions for *cox2* and 28S rRNA fragments followed the above except that the annealing temperatures were modified to 51.5 °C and 58.8 °C, respectively. The PCR products were purified and sequenced in both directions at Shanghai Sunny Biotechnology Co., Ltd (China).

### Sequence alignment, phylogenetic analyses and molecular dating

The nucleotide sequences of the seven species of *Cerapanorpa* are deposited in the GenBank database and their accession numbers are shown in Supplementary Table S1. We also obtained target sequences of 11 species of *Panorpa*, four of *Neopanorpa*, three of *Dicerapanorpa* and *Sinopanorpa*, two of *Cerapanorpa*, and one of *Furcatopanorpa*, *Panorpodes* and *Brachypanorpa* from the GenBank (see Supplementary Table S1). *Panorpodes kuandianensis* Zhong, Zhang & Hua, 2011 and *Brachypanorpa carolinensis* (Banks, 1905) of Panorpodidae were used as outgroups in the phylogenetic analysis. All three genes were aligned separately using MUSCLE^36^ with default parameter settings. The two mitochondrial genes were translated into amino acids in order to verify the desired protein-coding genes sequenced.

Partitioned Bayesian inference (BI) was performed in BEAST 1.8.4^37^ to reconstruct the phylogeny of Panorpidae and estimate the divergence times. The partitions and models of nucleotide substitution were selected under PartitionFinder 1.1.1^38^, using the “greedy” algorithm, the “beast” set of models and the Bayesian Information Criterion (BIC). The model of HKY + G was chosen for 28S rDNA and Yang96 for *cox1* and *cox2*. We analyzed the data under an uncorrelated log-normal relaxed clock and a Yule speciation process. Four runs were conducted with randomly generated starting trees and a chain length of 800 million generations, sampling every 100 generations. The stationarity and convergence of chains were checked in Tracer 1.6^39^ to ensure that effective sample sizes were greater than 200 for all parameters. A burn-in fraction of 50% was discarded in TreeAnnotator (part of the BEAST package) before exporting a maximum clade credibility tree. The final tree was visualized in FigTree 1.4.3 (available at http://tree.bio.ed.ac.uk/software/figtree).

Fossil data available were used to calibrate the phylogenetic tree. The oldest confident panorpids were from the Ypresian Okanagan Highlands^40^. Therefore, the time to the most recent ancestor (TMRCA) of *Panorpa* was defined in the middle Ypresian at 52.90 Ma ± 0.83 Ma based on specimens from McAbee, Canada^41^.

## Results

### Cytogenetic analysis

**Figure 1:**
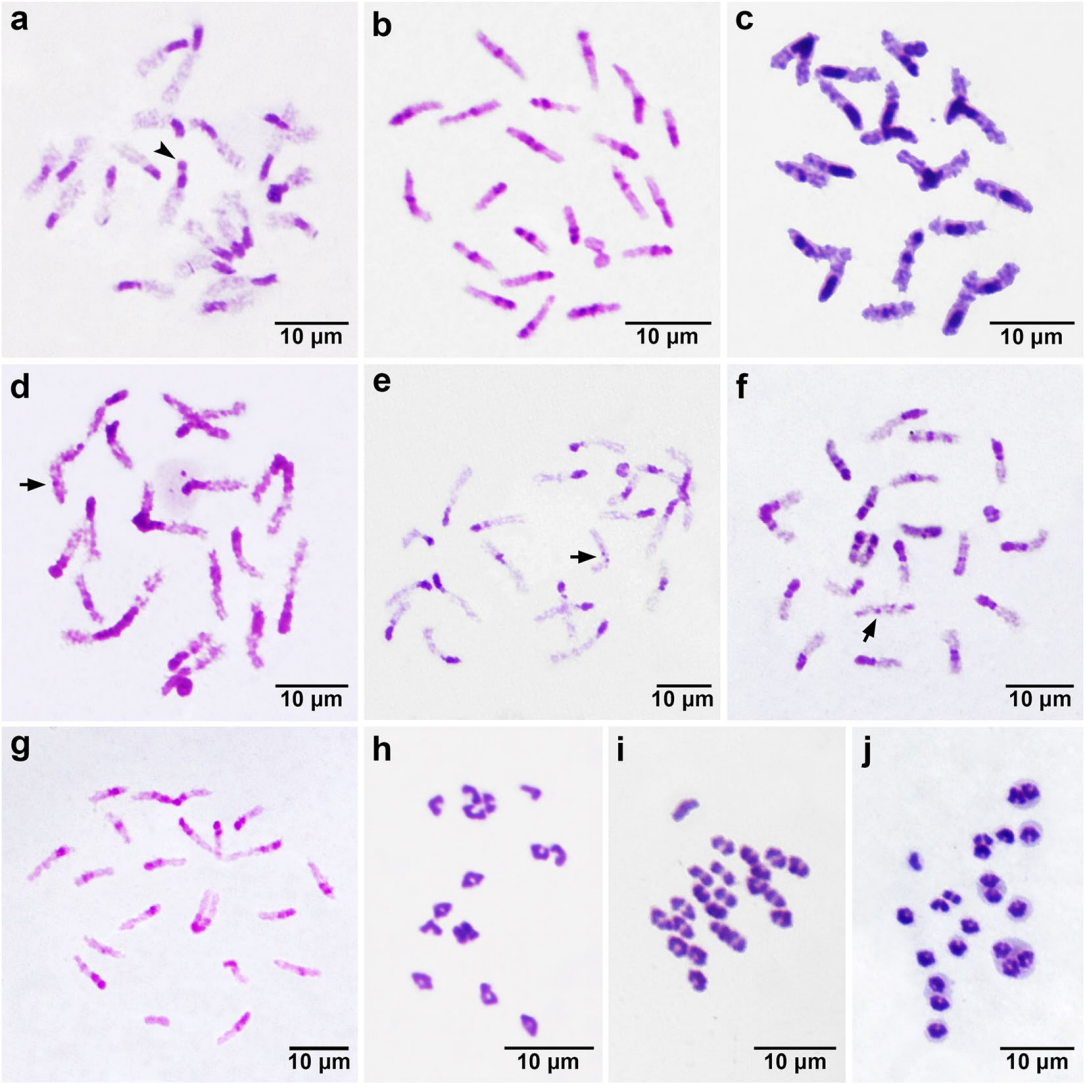
Bivalent spreading of *Cerapanorpa* spp. after C-banding at the condensation stage (**a**–**g**) and at metaphase I to anaphase I (**h**–**j**). (**a**,**h**) *C*. *brevicornis*; (**b**,**i**) *C*. *dubia*; (**c**,**j**) *C*. *byersi*; (**d**) *C*. *obtusa*; (**e**) *C*. *protrudens*; (**f**) *C*. *nanwutaina*; (**g**) *C*. *sinuata*. Arrows point to the heteromorphic bivalent; arrowhead points to the satellite chromosome.

All the males of *Cerapanorpa* species examined have the same chromosome number 2*n* = 43 and X0 sex determination (Fig. 1). The sizes of bivalents decrease gradually from pair to pair, and the bivalents are almost impossible to be grouped into different length classes. The sex univalent is morphologically variable at different stages during meiosis. The C-banding reveals a predominance of constitutive heterochromatin, although the patterns vary among the taxa. Conspicuous heterochromatin at one bivalent terminal is the most conserved type after treatment with the C-banding technique.

**Figure 2:**
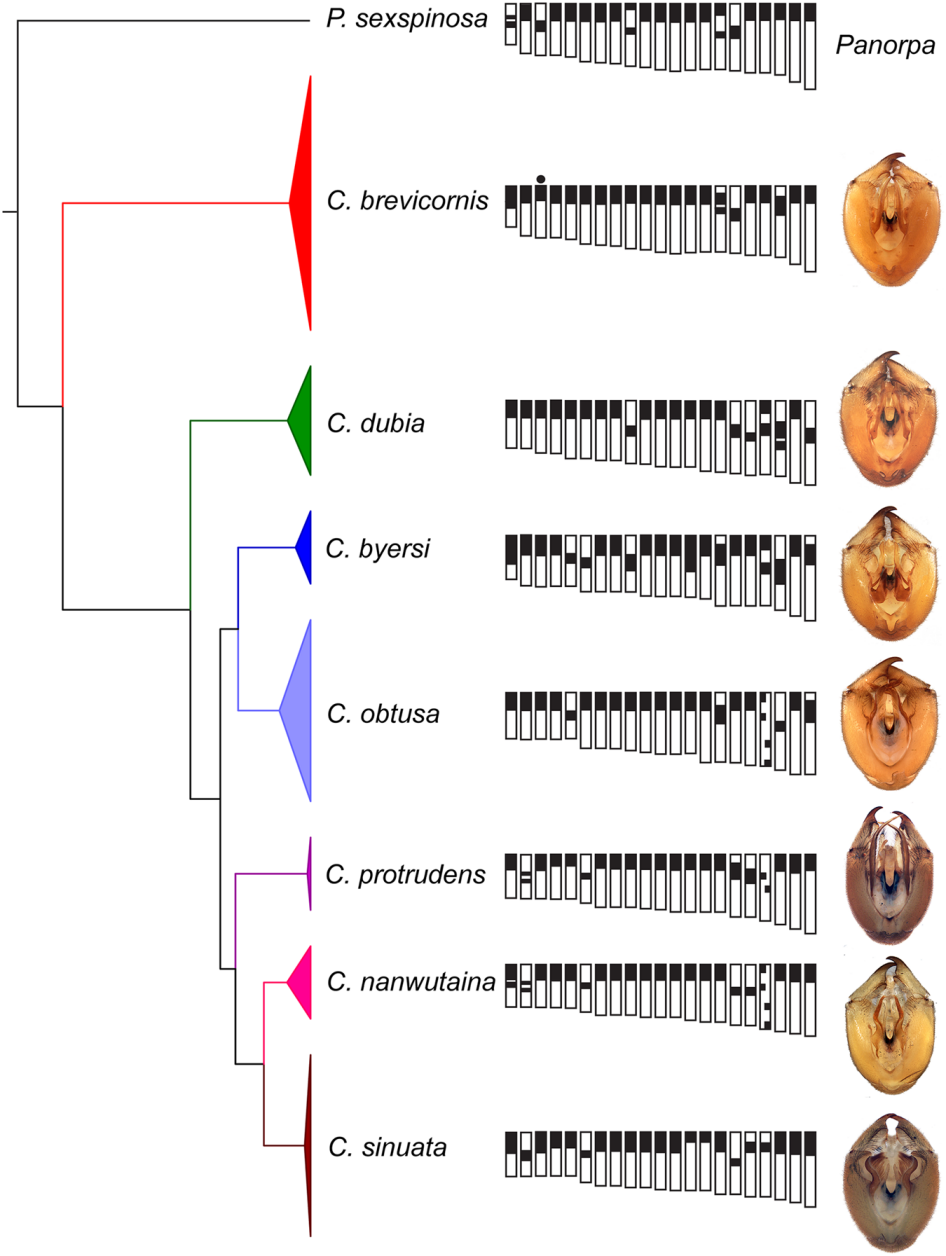
Phylogenetic relationship of the seven species in *Cerapanorpa* with the schematic representation of the main C-bands (black blocks) and the male genitalia in ventral views.

*Cerapanorpa brevicornis* is well characterized by a satellite chromosome (arrowhead in Fig. 1a) and a simple banding pattern (Fig. 2). Eighteen bivalents (from 3.89% ± 0.07% to 5.92% ± 0.29%, Table 1) exhibit terminal heterochromatin, two (4.98% ± 0.08% and 5.22% ± 0.11%) have subterminal heterochromatin, and only one (5.09% ± 0.08%) shows intermediate heterochromatin (Fig. 1a).

The C-banding pattern of *C*. *dubia* is represented by intermediate bands on five bivalents (from 4.54% ± 0.07% to 6.61% ± 0.47%), and two distant bands at terminal and intermediate regions on one bivalent (5.49% ± 0.13%) (Figs 1b and 2).

The banding type of *C*. *byersi* is similar to that of *C*. *dubia*, except that one bivalent exhibits a large sub-terminal block in *C*. *byersi* instead of a small median block in *C*. *dubia* (Figs 1c and 2). The heterochromatin occupies almost half of the chromosome length in *C*. *byersi*. In addition, *C*. *byersi* has one more bivalent with subterminal heterochromatin than *C*. *dubia*.

In *C*. *obtusa* only five bivalents exhibit varied bands. Two bivalents (4.98% ± 0.08% and 5.22% ± 0.11%) show intermediate bands. Two bivalents (4.98% ± 0.08% and 5.22% ± 0.11%) show sub-terminal bands. One bivalent (5.22% ± 0.11%) exhibits heteromorphy, with the homologues having asymmetric bands (arrow in Fig. 1d). Heteromorphic bivalents are also visible in *C*. *protrudens* and *C*. *nanwutaina* (arrows in Fig. 1e,f).

Of the 21 autosomal bivalents of *C*. *nanwutaina*, 16 bivalents (from 3.78% ± 0.40% to 6.21% ± 0.22%) exhibit conserved terminal heterochromatin, and five exhibit different C-patterns. Four bivalents (from 3.90% ± 0.32% to 5.60% ± 0.08%) show intermediate heterochromatin, and one bivalent (5.92% ± 0.01%) exhibits asymmetric bands (arrow in Fig. 1f).

The C-banding pattern of *C*. *protrudens* is roughly the same as that of *C*. *nanwutaina* except that one bivalent exhibits subterminal heterochromatin in *C*. *protrudens* (Fig. 1e) instead of intermediate heterochromatin as in *C*. *nanwutaina*.

*Cerapanorpa sinuata* exhibits intermediate blocks on three bivalents and subterminal blocks on two bivalents (Figs 1g and 2).

Among a variety of species, meta-anaphase bivalents exhibit varied morphology (Fig. 1h–j), which may be partially resulted from the different percentages of terminal heterochromatin on bivalents.

### Phylogeny of *Cerapanorpa*

**Figure 3:**
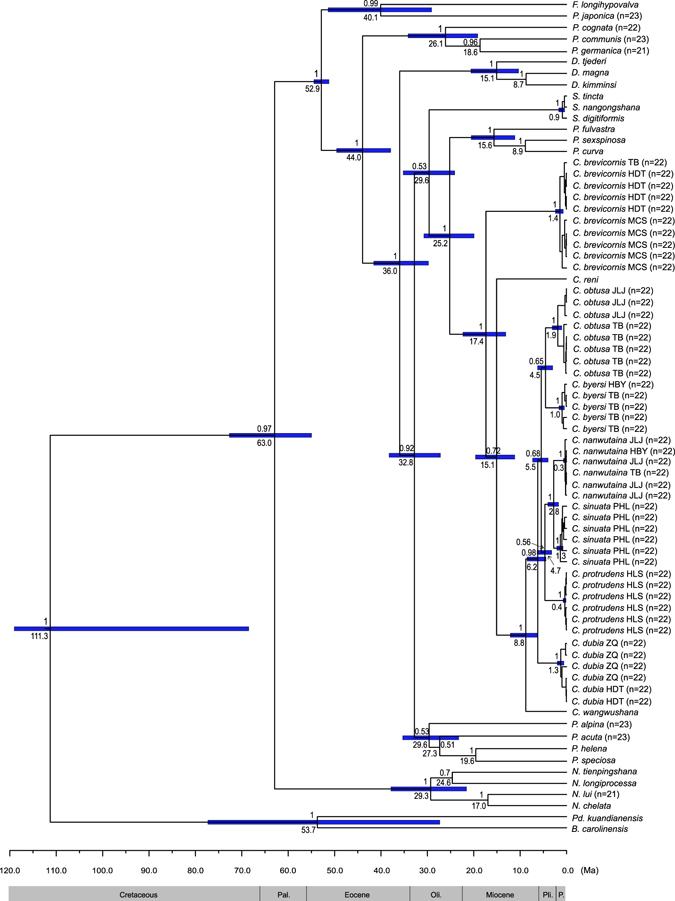
Bayesian maximum-clade-credibility tree based on the concatenated dataset (28S rRNA, *cox1* and *cox2*) in BEAST 1.8.4 with a relaxed clock and Yule speciation process. Node numbers above blue bars show Bayesian posterior probabilities (PP); node numbers below blue bars indicate the mean estimated divergence times of Panorpidae in million years ago (Ma). Horizontal blue bars at nodes represent 95% highest posterior density date ranges. Pal., Paleocene; Oli., Oligocene; Pli., Pliocene; P., Pleistocene.

An alignment of 1521 bp was obtained for the concatenated data of one nuclear gene 28S rRNA and two mitochondrial genes *cox1* and *cox2*, including 630 variable sites and 470 parsimony informative sites. The Bayesian consensus phylogeny based on the combined sequences shows that the monophyly of *Cerapanorpa* is well supported and can be grouped into two principal clades with a high support value (Bayesian posterior probability, PP = 1.0) (Fig. 3). The specimens of *C*. *brevicornis* form one clade. In this clade, the individuals from Micangshan (in the Bashan Mountains) form a subclade as a sister taxon to those from Taibai and Huoditang (both in the Qinling Mountains) with a strong support value (PP = 1.0).

The other clade comprises the remaining species of *Cerapanorpa* studied and is further subdivided into eight subclades, clearly showing that each species is an evolutionarily independent entity (Fig. 3). According to the topology of the cladogram, *Cerapanorpa reni* (Chou, 1981) forms a sister taxon to all the remaining species. *Cerapanorpa wangwushana* (Huang, Hua & Shen, 2004) has a sister group relationship with *C*. *dubia* + ((*C*. *obtusa* + *C*. *byersi*) + (*C*. *protrudens* + (*C*. *nanwutaina* + *C*. *sinuata*))). *Cerapanorpa protrudens* forms the sister species to *C*. *nanwutaina* + *C*. *sinuata*, and these three species together constitute the sister group to the subclade of *C*. *obtusa* + *C*. *byersi*.

### Divergence time estimation

The Bayesian time-calibrated tree infers that *Cerapanorpa* split from *Panorpa* at a mean age of 25.2 Ma (95% highest posterior density interval, HPD = 19.9–30.7 Ma, Fig. 3). The species of *Cerapanorpa* diverged from 17.4 Ma (95% HPD = 13.1–22.3 Ma). The estimated divergence time is 1.4 Ma (95% HPD = 0.7–2.4 Ma) between the two subclades of *C*. *brevicornis* from the Qinling and Bashan Mountains. *Cerapanorpa dubia* has a relatively recent origin at 6.2 Ma (95% HPD = 4.4–8.5 Ma). *Cerapanorpa byersi* diverged from *C*. *obtusa* at 4.5 Ma (95% HPD = 3.0–6.3 Ma). The TMRCA of *C*. *nanwutaina*, *C*. *sinuata* and *C*. *protrudens* is estimated at 4.7 Ma (95% HPD = 3.2–6.3 Ma), whereas *C*. *nanwutaina* and *C*. *sinuata* diverged from each other at around 2.8 Ma (95% HPD = 1.7–4.0 Ma).

## Discussion

In the present investigation, all species display similar cytogenetic features with 2*n* = 43 chromosomes, achiasmate meiosis and X0 male sex determination, reconfirming the monophyly of the genus *Cerapanorpa* as proposed by Hu *et al*.^27^ and Ma *et al*.^42^. These results are consistent with the uniform appearance of *Cerapanorpa*, which differs diagnostically from other groups of Panorpidae by the presence of a finger-like anal horn on the posterior margin of tergum VI in the males^20, 24^.

The anal horn is also found in the North American *Panorpa rufescens* group, the male tergum VI of which is produced posteriorly into a flat or conical projection^43–45^, which differs greatly in morphology from the digitate or finger-like anal horn in *Cerapanorpa* ^20, 24^. Cytogenetic differences in chromosome number between *Cerapanorpa* (2*n* = 43) and the *P*. *rufescens* group (2*n* = 45)^29^ strongly support Gao *et al*.’s viewpoint^20^ that the so-called anal horns are homoplasious characters between *Cerapanorpa* and the *P*. *rufescens* group. A recent molecular phylogeny of Panorpidae also shows that *Cerapanorpa* (as the *P*. *centralis* group previously) diverges from the North American species distantly^27^.

Our cytogenetic data are inconsistent with the previous reports that *C*. *dubia* and *C*. *emarginata* have the lowest chromosome number (2*n* = 39) in Panorpidae^28^. Their different counts of chromosome number may partly result from the frequent occurrences of end-to-end association, which caused non-homologous chromosomes to appear as a single unit in the Panorpidae^29^. Alternatively, the low quality of their figures in previous studies may also contribute to this miscount.

Great variations in the size and number of heterochromatic bands were observed in the species of *Cerapanorpa*, based on seven species (*ca*. 32% of the total species) analyzed. The patterns of C-bands exhibit a great interspecific variation and are constant within a species, implying that they play a substantial significance in species delimitation of the genus. *Cerapanorpa brevicornis* is characterized by only one bivalent with intermediate heterochromatin and the presence of a satellite chromosome, corresponding to its relatively distant relationships with other congeners in the phylogenetic tree (Fig. 3) and its very short anal horn and elongate parameres^24^ (Fig. 2).

The remaining species of *Cerapanorpa* exhibit an astonishing variability in C-banding, which differs evidently from that of *C*. *brevicornis*. *Cerapanorpa byersi* is characterized by large blocks of heterochromatin covering almost half of the bivalents. This heterochromatin amplification likely results from intensive retrotransposon activity or the concerted evolution of tandem repetitive DNA, especially following chromosome rearrangements^5, 6^. The C-banding pattern of *C*. *obtusa* is pronouncedly different from that of *C*. *byersi*. Although *C*. *obtusa* forms a sister taxon to *C*. *byersi*, the clade of *C*. *obtusa* + *C*. *byersi* receives a weak support (PP = 0.65 in Fig. 3) and is morphologically variable^24, 46^, suggesting a necessity for a detailed further taxonomic analysis.

Natural C-banding polymorphism was observed in *C*. *protrudens* and *C*. *nanwutaina*, implying that chromosomal rearrangement events play a significant role in generating differentiated banding type. The low frequency of asymmetric bands strongly suggests that the differences of heterochromatin between homologues likely affect the normal chromosome pairing. In achiasmate organisms, heterochromatin may act as a meiotic matchmaker^47^. Pairing between homologous chromosomes with different heterochromatin results in disjunction problems during meiosis and subsequently reduces fertility^48–50^. Therefore, karyotypic differences form a partial post-zygotic reproductive barrier^51^, and preclude gene flow^52, 53^.

*Cerapanorpa nanwutaina* and *C*. *sinuata* exhibit evident differences in C-banding patterns, but show high similarity in genetic data (Figs 2 and 3) and external morphology^20, 54^. It is interesting to note that both species occur in the Huoditang Forest Farm, but they do not overlap in habitats. The smaller *C*. *sinuata* (forewing length 12.0–13.0 mm in males)^20^ occupies a higher elevation (>2200 m) than the larger *C*. *nanwutaina* (forewing length 14.0 mm in males, elev. 1500–1650 m)^54^. The adaptation to increased elevation may result in a decline of body size in montane insects^55, 56^. Therefore, the two species are likely evolved from a common ancestor, which diverged into two species with preferences for different elevations and habitats.

The phylogeny reconstructed here is congruent with our cytogenetic findings, confirming that the closely related species of *Cerapanorpa* are evolutionarily independent entities. The specimens of *C*. *brevicornis* with a very short anal horn on male tergum VI are clustered into a well-supported clade, which forms a sister taxon to the remaining species of *Cerapanorpa* with a long anal horn on male tergum VI. This suggests a possible evolutionary trend of the anal horn, which is likely evolved from a virtual absence as found in most other panorpids toward a less-developed process as in *C*. *brevicornis*, and eventually to a well-developed and elongated digitate horn as in other species of *Cerapanorpa*.

*Cerapanorpa brevicornis* is widespread in the Bashan Mountains, but occurs only in scattered localities in the Qinling Mountains^24^. On the other hand, other species of *Cerapanorpa* studied herein are mainly distributed in the Qinling Mountains^20^. Based on our relaxed molecular clock analysis, the most diversification of *Cerapanorpa* occurred from the Burdigalian to the Piacenzian (17.4−2.8 Ma) in the Neogene (Fig. 3). During this period, the landscape of the Qinling-Bashan Mountains has been strongly influenced and remodeled due to the Meso-Cenozoic intracontinental orogeny^57–59^. A series of fault zones and foreland basins have been formed between the Qinling and the Bashan Mountains^59, 60^. Due to the relatively weak flight ability of Panorpidae and the forest fragmentation of the region, the dispersal of the Panorpidae are severely limited by fairly narrow zones of unsuitable habitats^18, 61^. The occurrence of geographical barriers, including numerous Bashan basins and the Hanshui River between the Qinling and the Bashan Mountains, may provide opportunity for the isolation of the populations and accumulation of chromosome mutations, which are likely responsible for the production of evolutionary novelties and eventually the generation of new taxa. The existence of the two clades of *C*. *brevicornis* between the Qinling and the Micangshan populations suggests that there occurs the complex outcome of several cycles of population vicariance, expansion and contraction. The inferred divergence of the two clades likely occurred in the Calabrian (*ca*. 1.4 Ma), suggesting the succession of the Pleistocene glacial-interglacial cycles seems to be the cause of numerous range shifts in *C*. *brevicornis* ^62–64^.

Our present study demonstrates that cytogenetic data may play a significant role in the species delimitation and the speciation process of *Cerapanorpa*. Admittedly, we chose only approximately one-third of the total species of *Cerapanorpa* for the cytogenetic study because the chromosome research needs live specimens that should happen to be in the suitable physiological period of spermatogenesis of the males. It is necessary to add more species and more individuals from a variety of sampling sites to make the conclusions more robust for future studies in *Cerapanorpa*. Application of additional markers should be another choice.

## Electronic supplementary material

Supplementary Table S1
