# Supplementary material for: Cytotaxonomy and molecular phylogeny of the genus Cerapanorpa Gao, Ma & Hua, 2016 (Mecoptera: Panorpidae)
Source: Sci Rep. 2017 Jul 3;7:4493. doi: 10.1038/s41598-017-04926-9 (PMC5495786; doi:10.1038/s41598-017-04926-9)
Supplement: Supplementary file 1 — Supplementary Table S1 [file 41598_2017_4926_MOESM1_ESM.pdf]

Cytotaxonomy and molecular phylogeny of the genus *Cerapanorpa* Gao, Ma & Hua, 2016 (Mecoptera: Panorpidae)

Ying Miao[1], Na Ma[1,2,*] & Bao-Zhen Hua[1,*]

Table S1 List of species for phylogenetic reconstruction and their related information.

Species	Locality	GenBank accession Nos. for *cox1*, *cox2* and 28S	Collecting date/ source
**Panorpidae**			
Cerapanorpa brevicornis (Hua & Li, 2007)	Huoditang (HDT), Shaanxi, 1650 m	KY582997; KY583030; KY582963	vi. 2013
C. brevicornis	Huoditang (HDT), Shaanxi, 1650 m	KY582998; KY583031; KY582964	vi. 2013
C. brevicornis	Huoditang (HDT), Shaanxi, 1650 m	KY582999; KY583032; KY582965	vi. 2013
C. brevicornis	Huoditang (HDT), Shaanxi, 1650 m	MF068767; MF068788; MF068746	vi. 2013
C. brevicornis	Micangshan (MCS), Shaanxi, 1600 m	KY583000; KY583033; KY582966	vi. 2013
C. brevicornis	Micangshan (MCS), Shaanxi, 1600 m	KY583001; KY583034; KY582967	vi. 2013
C. brevicornis	Micangshan (MCS), Shaanxi, 1600 m	KY583002; KY583035; KY582968	vi. 2013
C. brevicornis	Micangshan (MCS), Shaanxi, 1600 m	KY583003; KY583036; KY582969	vi. 2013
C. brevicornis	Micangshan (MCS), Shaanxi, 1600 m	MF068768; MF068789; MF068747	vi. 2013
C. brevicornis	Taibai (TB), Shaanxi, 1800 m	KY583004; KY583037; KY582970	vi. 2013
C. byersi (Hua & Huang, 2007)	Taibai (TB), Shaanxi, 2100 m	KY583005; KY583038; KY582971	vi. 2014
C. byersi	Taibai (TB), Shaanxi, 2100 m	KY583006; KY583039; KY582972	vi. 2014
C. byersi	Taibai (TB), Shaanxi, 2100 m	KY583007; KY583040; KY582973	vi. 2014
C. byersi	Taibai (TB), Shaanxi, 2100 m	MF068764; MF068785; MF068743	vi. 2014
C. byersi	Huangboyuan (HBY), Shaanxi, 1500 m	MF068765; MF068786; MF068744	vi. 2014
C. dubia (Chou & Wang, 1981)	Zhuque (ZQ), Shaanxi, 1390 m	KY583008; KY583041; KY582974	vi. 2014
C. dubia	Zhuque (ZQ), Shaanxi, 1390 m	KY583009; KY583042; KY582975	vi. 2014
C. dubia	Zhuque (ZQ), Shaanxi, 1390 m	KY583010; KY583043; KY582976	vi. 2014
C. dubia	Zhuque (ZQ), Shaanxi, 1390 m	KY583011; KY583044; KY582977	vi. 2014
C. dubia	Huoditang (HDT), Shaanxi, 1650 m	MF068769; MF068790; MF068748	vi. 2015

Species	Locality	GenBank accession Nos. for *cox1*, *cox2* and 28S	Collecting date/ source
C. dubia	Huoditang (HDT), Shaanxi, 1650 m	MF068770; MF068791; MF068749	vi. 2015
C. nanwutaina (Chou, 1981)	Jialingjiang (JLJ), Shaanxi, 1500 m	KY583012; KY583045; KY582978	vi. 2014
C. nanwutaina	Jialingjiang (JLJ), Shaanxi, 1500 m	KY583013; KY583046; KY582979	vi. 2014
C. nanwutaina	Jialingjiang (JLJ), Shaanxi, 1500 m	KY583014; KY583047; KY582980	vi. 2014
C. nanwutaina	Huangboyuan (HBY), Shaanxi, 1500 m	KY583015; KY583048; KY582981	vi. 2014
C. nanwutaina	Jialingjiang (JLJ), Shaanxi, 1500 m	KY583016; KY583049; KY582982	vi. 2014
C. nanwutaina	Taibai (TB), Shaanxi, 1800 m	KY583017; KY583050; KY582983	vi. 2014
C. obtusa (Cheng, 1949)	Taibai (TB), Shaanxi, 2100 m	KY583018; KY583051; KY582984	vi. 2014
C. obtusa	Taibai (TB), Shaanxi, 2100 m	KY583019; KY583052; KY582985	vi. 2015
C. obtusa	Taibai (TB), Shaanxi, 2100 m	KY583020; KY583053; KY582986	vi. 2014
C. obtusa	Taibai (TB), Shaanxi, 2100 m	KY583021; KY583054; KY582987	vi. 2014
C. obtusa	Taibai (TB), Shaanxi, 2100 m	MF068773; MF068794; MF068752	vi. 2014
C. obtusa	Jialingjiang (JLJ), Shaanxi, 1500 m	KY583022; KY583055; KY582988	vi. 2014
C. obtusa	Jialingjiang (JLJ), Shaanxi, 1500 m	KY583023; KY583056; KY582989	vi. 2014
C. obtusa	Jialingjiang (JLJ), Shaanxi, 1500 m	MF068771; MF068792; MF068750	vi. 2014
C. protrudens Gao, Ma & Hua, 2016	Hualongshan (HLS), Shaanxi, 2100 m	KY583024; KY583057; KY582990	vi. 2015
C. protrudens	Hualongshan (HLS), Shaanxi, 2100 m	KY583025; KY583058; KY582991	vi. 2015
C. protrudens	Hualongshan (HLS), Shaanxi, 2100 m	KY583026; KY583059; KY582992	vi. 2015
C. protrudens	Hualongshan (HLS), Shaanxi, 2100 m	MF068776; MF068797; MF068755	vi. 2015
C. protrudens	Hualongshan (HLS), Shaanxi, 2100 m	MF068777; MF068798; MF068756	vi. 2015
C. protrudens	Hualongshan (HLS), Shaanxi, 2100 m	MF068778; MF068799; MF068757	vi. 2015

**Species**	**Locality**	**GenBank accession Nos. for *cox1*, *cox2* and 28S**	**Collecting date/ source**
C. sinuata Gao, Ma & Hua, 2016	Pingheliang (PHL), Shaanxi, 2200 m	KY583027; KY583060; KY582993	vii. 2015
C. sinuata	Pingheliang (PHL), Shaanxi, 2200 m	KY583028; KY583061; KY582994	vii. 2015
C. sinuata	Pingheliang (PHL), Shaanxi, 2200 m	KY583029; KY583062; KY582995	vii. 2015
C. sinuata	Pingheliang (PHL), Shaanxi, 2200 m	MF068781; MF068802; MF068760	vii. 2015
C. sinuata	Pingheliang (PHL), Shaanxi, 2200 m	MF068782; MF068803; MF068761	vii. 2015
C. sinuata	Pingheliang (PHL), Shaanxi, 2200 m	MF068783; MF068804; MF068762	vii. 2015
C. reni (Chou, 1981)	China	MF002437; MF002438; MF002439	vi. 2015
C. wangwushana (Huang, Hua & Shen, 2004)	China	GU722384; GU722400; HM061591	Hu et al. (2015)
Dicerapanorpa kimminsi (Carpenter, 1948)	China	JQ011461; KJ816713; JQ011462	Hu et al. (2015)
D. magna (Chou, 1981)	China	GU722385; GU722401; HM061594	Hu et al. (2015)
D. tjederi (Carpenter, 1938)	China	KJ816730; KJ816696; KJ816714	Hu et al. (2015)
Furcatopanorpa longihypovalva (Hua & Cai, 2009)	China	GU722391; GU722407; HM061600	Hu et al. (2015)
Neopanorpa chelata Carpenter, 1938	China	GU722395; GU722412; HM061598	Hu et al. (2015)
N. longiprocessa Hua & Chou, 1997	China	GU722387; GU722403; HM061597	Hu et al. (2015)
N. lui Chou & Ran, 1981	China	KJ816732; KJ816698; KJ816716	Hu et al. (2015)
N. tienpingshana Chou & Wang, 1988	China	KJ816737; KJ816703; KJ816721	Hu et al. (2015)
Panorpa acuta Carpenter,1931	USA	AF180070; AF424033; AF423967	Misof et al. (2000) and Whiting (2002)
P. alpina Rambur, 1842	Germany	AF180069; KY583063; KY582996	vi. 2016
P. cognata Rambur, 1842	Europe	AF180072; KJ816712; AF423954	Misof et al. (2000) and Whiting (2002)
P. communis Linnaeus, 1758	Europe	AF180071; AF424024; AF423957	Misof et al. (2000) and Whiting (2002)
P. germanica Linnaeus, 1758	Europe	AF180075; AF424032; AF423965	Misof et al. (2000) and Whiting (2002)

**Species**	**Locality**	**GenBank accession Nos. for *cox1*, *cox2* and 28S**	**Collecting date/ source**
P. helena Byers, 1962	USA	AF180076; AF424029; AF338264	Misof et al. (2000) and Whiting (2002)
P. japonica Thunberg, 1784	Japan	EF050552; EF050564; AF423969	Pollmann et al. (2008) and Whiting (2002)
P. speciosa Carpenter, 1931	USA	KJ816743; KJ816709; KJ816727	Hu et al. (2015)
Sinopanorpa digitiformis Huang & Hua, 2008	China	JN688131; JN688142: JN688119	Hu et al. (2015)
S. nangongshana Cai & Hua, 2008	China	JN688127; JN688141; JN688120	Hu et al. (2015)
S. tincta (Navás, 1931)	China	GU722388; GU722404; HM061595	Hu et al. (2015)
**Panorpodidae**			
Panorpodes kuandianensis Zhong, Zhang & Hua, 2011	China	JN223516; JN223498; JN223487	Hu et al. (2015)
Brachypanorpa carolinensis (Banks, 1905)	USA	EF050557, EF050569, EF050542	Pollmann et al. (2008)
